# Microbial Genetics and Evolution

**DOI:** 10.3390/microorganisms10071274

**Published:** 2022-06-23

**Authors:** Sara Del Duca, Alberto Vassallo, Alessio Mengoni, Renato Fani

**Affiliations:** 1 Department of Biology, University of Florence, 50019 Sesto Fiorentino, Italy; sara.delduca@unifi.it (S.D.D.); alessio.mengoni@unifi.it (A.M.); 2 School of Biosciences and Veterinary Medicine, University of Camerino, 62032 Camerino, Italy; alberto.vassallo@unicam.it

Although proto-evolutionary ideas date back to the time of the ancient Greeks, the idea that organisms evolve was not considered a basic element of scientific knowledge until Charles Darwin published his “On the Origin of Species” in 1859. Darwin argued that all the organisms on the planet emerged through a single, straightforward process that operated relentlessly from the dawn of life and that continues to shape the natural world [1]. 

*Microorganisms* have been thriving on Earth for billions of years, and their environments have undergone fundamental changes over this time: thus, they have evolved extraordinary capacities to adapt to environmental and evolutionary challenges. Microbial genomes are highly dynamic: the acquisition of foreign DNA combined with intra-genomic rearrangement and duplication events may provide an explanation for the remarkable ability of microorganisms to constantly explore new ecological niches [2]. The use of microbes to experimentally address evolutionary questions became routine more than three decades ago [3]: thanks to their short generation times and large population sizes, microbes evolve rapidly [4], rendering them extraordinary models to study evolution in action and its genetic basis. 

*Microorganisms* display extraordinary variation in their metabolic properties, cellular structures, and lifestyles. Even within relatively narrow taxonomic groups, the phenotypic diversity among species is remarkable [5]. Clues as to how they started and traveled along this evolutionary road are conserved in their genomes; the current structure and organization of genes and genomes have been increasingly characterized thanks to modern and efficient molecular techniques, making possible a better understanding of the mechanisms at the basis of their evolution, expansion, and shaping.

Several molecular mechanisms may have been responsible for the expansion and shaping of genomes and, thus, for the evident differences among microbial species; these include internal mechanisms such as point mutations (substitutions, insertions, deletions) leading to the modification, inactivation, or differential regulation of existing genes [5], gene elongation, loss, duplication and/or fusion, and external mechanisms, such as cell fusion (synology) and horizontal gene transfer (xenology) [6,7]. The distribution of metabolic routes between microorganisms might have occurred through xenology or synology events [6]. The acquisition of new genetic material could have also been achieved by the internal duplication of DNA regions, whilst genes and genomes shaping to develop new functions (generally gained through evolutionary divergence) could have been satisfied thanks to the other cited internal molecular mechanisms [8].

The diversity observed in the microbial world allows the comprehension of the processes behind genetic diversity; bacteria, archaea, viruses, and yeasts are the subjects of laboratory studies and in silico analyses, aiming to investigate the evolution of their genomes and the link between genotypes and phenotypes. 

This Special Issue gathers eight research papers related to microbial genetics and evolution. A crucial step in investigating the evolution of an organism and its genome is to define its phylogeny: the introduction of DNA sequencing techniques and the ever-increasing availability of completely sequenced genomes allowed, during the last years, the emergence of comparative analyses of a huge number of genes and genomes [9]. The combination of data from genomic and evolutionary studies gave rise to the *phylogenomics* approach, which uses phylogenetic principles to make sense of genomic data [10], and which main issues are (i) using molecular data to infer species relationships and (ii) using the available information on a species evolutionary history to gain insights into the mechanisms of molecular evolution [11]. In this Special Issue, five studies address the topic of phylogenetic analysis to better investigate the origin, organization, and distribution of bacterial biosynthetic genes and to elucidate the phylogenesis, ecology, and evolution of microbial taxonomic groups. Specifically, the structure, organization, and phylogenetic distribution of histidine biosynthetic genes were analyzed for the members of the Bacteroidota-Rhodothermota-Balneolota-Chlorobiota superphylum—a group of phylogenetically close bacteria with different surviving strategies—revealing different organizations of the genes and the involvement of many evolutionary mechanisms in the shaping of the *his* gene structure in this taxonomic group [12]. In another paper, the distribution of the azurin gene—coding for a protein of relevant interest because of its anticancer activity—was evaluated in the three domains of life. The observed patchy distribution of this gene suggested a possible loss of this gene during the evolution of several bacterial phyla and/or an ancient horizontal transfer [13]. Comparative genome analyses have also been performed to better characterize microorganisms belonging to the bacterial *Aminobacter* genus [14], *Vibrio parahaemolyticus* isolates from seafood [15], and methanogenic archaea [16]. Different genetic markers have been used to assess their phylogeny, e.g., clustered regularly interspaced short palindromic repeats (CRISPR), 16S rRNA genes, or the whole genome. All of these studies allowed a better understanding of the phylogenetic history of the considered organisms/genes, proposing possible molecular rearrangements for their evolution. In particular, all of these studies highlighted the importance of horizontal gene transfer as a fundamental evolutionary molecular mechanism. 

However, nowadays, it is known that mutation is the ultimate source of all genetic variation and provides the fuel for evolution [4]. Two studies of this Special Issue aimed to investigate genomic changes over time in model organisms such as *Escherichia coli* and the ciliate *Paramecium tetraurelia*. In Vitali et al. [17], *P. tetraurelia* was used to study the consequences of up to fifty amitotic divisions at the genome level and to provide an approach for investigating unequal chromosome segregation in polyploid cells. They demonstrated that somatic assortment in *P. tetraurelia* occurs rather slowly, being prevented by the high ploidy of its macronucleus. In the second work, a histidine-auxotrophic mutant strain of *E. coli*, having a single-nucleotide deletion in its *hisF* gene, was used to study the effects of selective pressure on the reversion of frameshift mutations through a directed-evolution experiment [18]. The frequency of revertant mutants and the kind of mutation were finally correlated to the intensity of selective pressure, cultivation time, the tertiary structure of HisF, and ability to grow in the absence of His.

The understanding of the genetic and ecological processes associated with the different microorganisms and their genomes, and the study of their metabolic potential, can also open new intriguing perspectives for their possible biotechnological use. Indeed, Artuso et al. [14] proposed that *Aminobacter* species could be exploited in bioaugmentation and bioremediation processes, while Gammuto et al. [13] suggested a possible development of new tools based on the use of *P. aeruginosa* azurin p28 domain for treating cancer diseases. Finally, in another work comprised in this Special Issue, Choi et al. [19] engineered the yeast *Saccharomyces cerevisiae* by modifying the expression of three peroxisome proliferation-related proteins: the obtained mutant strains had an increased amount of peroxisomes and/or enlarged peroxisomes that provided additional storage space for the accumulation of protopanaxadiol synthesized through the expression of heterologous biosynthetic genes.

Altogether, the works published in this Special Issue demonstrated once more how both prokaryotic and eukaryotic microorganisms could be used as powerful tools for the study of genetic evolution and how information about these models can be applied in a wide range of fields spanning from medicine to biotechnology.
